# Iron deposition and increased alveolar septal capillary density in nonfibrotic lung tissue are associated with pulmonary hypertension in idiopathic pulmonary fibrosis

**DOI:** 10.1186/1465-9921-11-37

**Published:** 2010-04-14

**Authors:** Kyung-Hee Kim, Fabien Maldonado, Jay H Ryu, Patrick W Eiken, Thomas E Hartman, Brian J Bartholmai, Paul A Decker, Eunhee S Yi

**Affiliations:** 1 Department of Pathology, Chungnam National University, School of Medicine, Daejeon, Korea; 2 Department of Medicine, division of Pulmonary and Critical Care Medicine, Mayo Clinic, Rochester, Minnesota, USA; 3 Department of Radiology, Mayo Clinic, Rochester, Minnesota, USA; 4 Division of Biomedical Statistics and Informatics, Mayo Clinic, Rochester, Minnesota, USA; 5 Department of Laboratory Medicine and Pathology, division of Anatomic Pathology Mayo Clinic, Rochester, Minnesota, USA

## Abstract

**Background:**

Early diagnosis of pulmonary hypertension (PH) in idiopathic pulmonary fibrosis (IPF) has potential prognostic and therapeutic implications but can be difficult due to the lack of specific clinical manifestations or accurate non-invasive tests. Histopathologic parameters correlating with PH in IPF are also not known. Remodeling of postcapillary pulmonary vessels has been reported in the nonfibrotic areas of explanted lungs from IPF patients. We hypothesized that iron deposition and increased alveolar capillaries, the findings often seen in postcapillary PH, might predict the presence of clinical PH, independent of the severity of fibrosis or ventilatory dysfunction in IPF patients. To test this hypothesis, we examined the association between these histologic parameters and the degree of PH, with consideration of the severity of disease in IPF.

**Methods:**

Iron deposition and alveolar septal capillary density (ASCD) were evaluated on histologic sections with hematoxylin-eosin, iron, elastin and CD34 stainings. Percentage of predicted forced vital capacity (FVC%) was used for grading pulmonary function status. Fibrosis score assessed on high resolution computed tomography (HRCT) was used for evaluating overall degree of fibrosis in whole lungs. Right ventricular systolic pressure (RVSP) by transthoracic echocardiography was used for the estimation of PH. Univariate and multivariate regression analyses were performed.

**Results:**

A cohort of 154 patients was studied who had the clinicopathological diagnosis of IPF with surgical lung biopsies or explants during the period of 1997 to 2006 at Mayo Clinic Rochester. In univariate analysis, RVSP in our IPF cases was associated with both iron deposition and ASCD (p < 0.001). In multivariate analysis with FVC% and HRCT fibrosis score included, iron deposition (p = 0.02), but not ASCD (p = 0.076), maintained statistically significant association with RVSP. FVC% was associated with RVSP on univariate analysis but not on multivariate analysis, while fibrosis score lacked any association with RVSP by either univariate or multivariate analyses.

**Conclusions:**

Iron deposition and ASCD in non fibrotic lung tissue showed an association with RVSP, suggesting that these features are possible morphologic predictors of PH in IPF.

## Background

Idiopathic pulmonary fibrosis (IPF) is characterized by a histopathologic pattern of usual interstitial pneumonia (UIP) and progressive fibrosis without response to medical therapy [1]. However, clinical course of IPF is not always predictable despite its generally poor prognosis [2]. Pulmonary hypertension (PH) has been postulated to be a factor that might complicate and impact the prognosis of IPF [3]. Also, the therapeutic agents for PH might be effective for those who have PH with IPF.

Early diagnosis of PH in IPF is difficult; lack of specific clinical symptoms often leads to delayed diagnosis of PH in IPF patients [3]. Reported prevalence of PH in IPF patients ranges from 20 to 84% when evaluated by pulmonary arterial enlargement on chest radiography, right ventricular systolic pressure (RVSP) using transthoracic echocardiography, or mean pulmonary artery pressure using right-heart catheterization [3-8]. Biomarkers such as B-type natriuretic peptide (BNP) and N-terminal prohormone BNP are potentially helpful tools in identifying PH [9].

Histologic features correlating with PH in IPF are not known. We hypothesized that increased alveolar septal capillaries and hemosiderin deposition, the findings often seen in the setting of postcapillary type of PH or pulmonary venous remodeling, might predict the presence of clinical PH independent of the degree of overall fibrosis or pulmonary function abnormalities in IPF cases.

In order to examine this hypothesis, we evaluated a cohort of 154 IPF cases for the association between these two histologic parameters and the right ventricular systolic pressure (RVSP) assessed by transthoracic echocardiography, with the consideration for the ventilatory function status as assessed by percentage of predicted forced vital capacity (FVC%) and for the extent of fibrosis by fibrosis score on chest high-resolution computed tomography (HRCT).

## Materials and methods

### Case selection

All patients who had a diagnosis of UIP by surgical lung biopsy (n = 144) or by examination of explanted lungs (n = 10) were identified during the period from 1997 to 2006. Patients with underlying collagen vascular disease or other identifiable cause for pulmonary fibrosis were excluded. A review of medical records was done for all cases for pertinent clinical information. This study has been approved by the Institutional Review Board of Mayo Foundation.

### Echocardiography

Two-dimensional transthoracic echocardiography, pulsed and continuous-wave Doppler, and color flow imaging were performed in all patients using previously described techniques [6]. Right ventricular systolic pressure was calculated based on the modified Bernoulli equation and right atrial pressure was estimated as 5, 10, 15 or 20 mm Hg, on the basis of the size and respiratory change of the inferior vena cava using previously described techniques [6]. Right ventricular size and systolic function were scored semiquantitatively on a scale 0 (normal), 1 (mildly impaired or enlarged), 2 (moderately impaired or enlarged), or 3 (severely impaired or enlarged). Left ventricular dimensions and ejection fraction were obtained by previously recommended techniques and scored semiquantitatively on a scale 0 (normal), 1 (mildly impaired or enlarged), 2 (moderately impaired or enlarged), or 3 (severely impaired or enlarged) [6]. Left atrial size was scored semiquantitatively on a scale 0 (normal), 1 (mildly enlarged), 2 (moderately enlarged), or 3 (severely enlarged).

### Pulmonary function test

Spirometry was performed using a Puritan Bennett renaissance pneumotach-based flow spirometer (Mallinckrodt, St Louis, MO, USA) according to the standards set by the American Thoracic Society/European respiratory Society (ATS/ERS) and flows were expressed as percentage of predicted using the reference equations of Crapo et al [10].

### Fibrosis score

Preoperative chest HRCT scans that were performed at the closest time point to the biopsy date or transplantation date were evaluated. If preoperative CT was not available, the earliest chest HRCT scans following the biopsies were reviewed. Each case was reviewed independently by two thoracic radiologists (PWE and BJB or TEH) and the final scores and measurements were assigned by consensus. Percentage of pulmonary parenchyma replaced by fibrosis was assigned based on visual estimation as previously described [11]. Each lung was evaluated for percentage fibrosis that was assigned to the nearest 5%.

### Histopathologic evaluation

In all surgical lung biopsy cases, entire available gross specimens were submitted for microscopic examination. For explanted lungs, multiple sections were reviewed from each lobe. All cases were evaluated by two pulmonary pathologists (KHK and ESY) and the diagnosis of UIP was confirmed according to the criteria outlined in ATS/ERS International Multidisciplinary Consensus Classification of the Idiopathic Interstitial Pneumonias [1,12]. Verhoeff-van Gieson elastic (VVG), iron, and CD34 stains as well as routine hematoxylin-eosin (HE) were used for microscopic evaluation of iron deposition and ASCD.

### Measurement of alveolar septal capillary density (ASCD)

**Figure 1:**
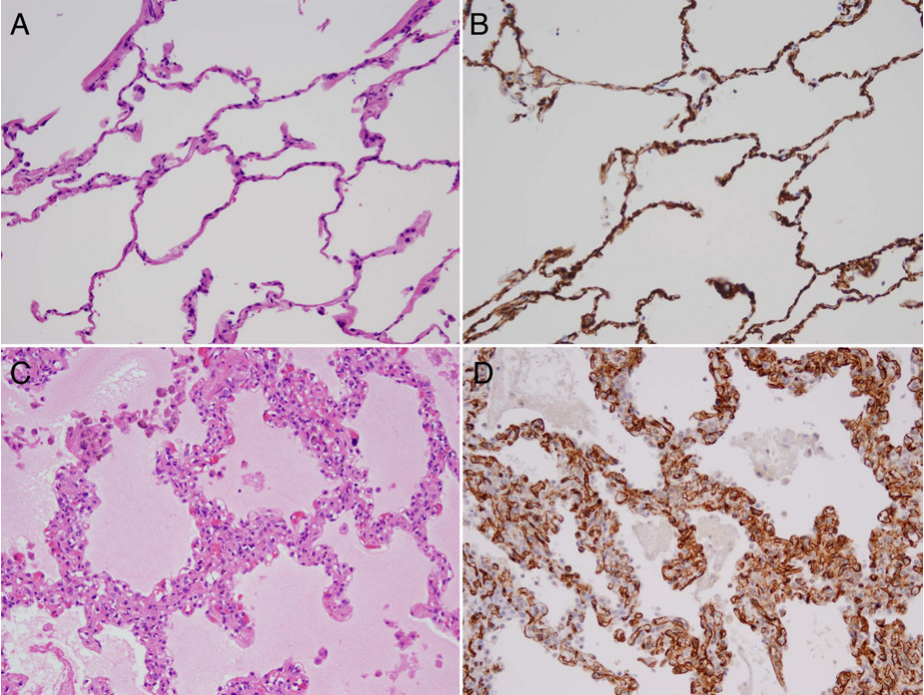
**A case showing grade 1 alveolar septal capillary density (ASCD) (A and B) without any increase in endothelial cells in the alveolar septa (A: hematoxylin eosin stain; B: anti CD34 immunostain, ×200 original magnification)**. A case showing grade 2 ASCD (C and D) with a marked increase in endothelial cells (C: hematoxylin eosin stain; D: anti CD34 immunostain, ×200 original magnification).

Sections were scanned for representative areas in nonfibrotic parenchyma at low-power field (×100 magnification), and individual alveolar capillary counts were then performed on high-power fields (×400 magnification) using CD34-stained sections. ASCD was quantified in representative alveolar capillary spots in ten nonfibrotic lobules. ASCD was expressed by the mean of capillary endothelial row between the alveolar epithelial layers in these ten nonfibrotic lobules of alveolar tissue, and categorized as Grade 1 and 2 with the mean of ASCD <1.5 and ≥1.5, respectively (Figure 1). This categorization as normal (ASCD <1.5; grade 1) and abnormal (ASCD ≥ 1.5; grade 2) is based on the fact that there should not be more than one row of capillaries between the alveolar septum with consideration tangential section [13].

### Assessment of hemosiderin deposition

**Figure 2:**
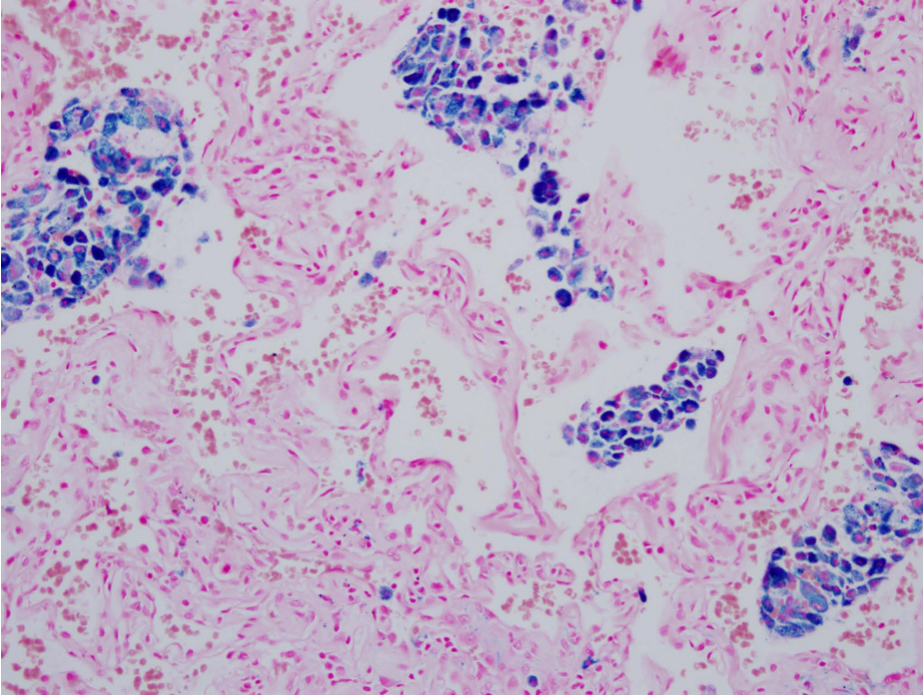
**A case with grade 3 iron deposition mainly involving the alveolar spaces (Prussian blue iron stain, ×200 original magnification)**.

Sections were scanned for hemosiderin deposition by the evaluation of iron staining at low power field (×100 magnification), and confirmed at high power fields (×400 magnification) to verify the presence of chunky, refractile hemosiderin pigments. Hemosiderin deposition was quantified as the total number of iron-positive foci on one representative glass slide and assigned to one of the following categories: 0, <1; 1, 1~5; 2, 6~10; and 3, >10 (Figure 2). This grading system is a modification from a previous study [14].

### Statistics

Continuous variables were summarized using mean ± standard deviation, median, and range; categorical variables were summarized using number and percentage. Univariable and multivariable regression analyses were performed to investigate the association of all variables with RVSP. For these analyses RVSP was the dependent variable. P-values < 0.05 were considered statistically significant.

## Results

Age of the patients ranged from 28 to 87 years (mean ± standard deviation: 64.4 ± 10.1 years; median: 66 years) (Table 1). Female and male patients comprised 40.3% and 59.7%, respectively (Table 1). There was no association between age and iron deposition or age and ASCD (Spearman's correlation coefficients -0.14 [p = 0.099) and -0.13 [p = 0.16], respectively).

**Table 1:** Demographics of patients

Variable	N	%
Age		
Mean ± standard deviation	64.4 ± 10.1	
Median; range	66; 28-87	
Gender		
Female	62	40.3
Male	92	59.7

Assessment of iron deposition was possible in 149 cases of the cohort and showed the following results: grade 0 in 67 cases, grade 1 in 41 cases, grade 2 in 24 cases and grade 3 in 17 cases. ASCD was possible to be assessed in 124 cases that had sufficient amount of evaluable non-fibrotic lung tissue. 75 cases showed grade 1 and 43 cases showed grade 2 of ASCD. FVC % within 6 months prior to biopsy was available in 146 patients and ranged from 26 to 109% of predicted value. Fibrosis score was possible to assess in 140 patients ranged from 10 to 195. 119 patients underwent transthoracic echocardiography with available RVSP that ranged from 24 to 114 mmHg. The numbers of patients having available RVSP data as well as iron deposition, ASCD, fibrosis score and FVC% were 114, 91, 116 and 113 cases, each.

The median interval between echocardiography and surgical lung biopsy was 14.5 days (range, 0-826 days); 75.4% of the echocardiograms had been performed within 3 months of the surgical lung biopsy. Left ventricular function was graded normal (score 0) in 93.2% and mildly impaired or enlarged (score 1) in 6.8% of patients. Left atrial size was normal (score 0) in 52.5%, mildly enlarged (score 1) in 31.4%, moderately enlarged (score 2) in 11.9% or severely enlarged (score 3) in 4.2% patients. Right ventricular function was normal (score 0) in 78%, mildly impaired or enlarged (score 1) in 11%, moderately impaired or enlarged in 8.5% and severely impaired or enlarged (score 3) in 2.5% of patients. There was significant association neither between iron deposition and left ventricular function, left atrial size or right ventricular function (Spearmans' correlation coefficients -0.06 [p = 0.55], 0.18 [p = 0.06], and 0.15 [p = 0.12], respectively), nor between ASCD and left ventricular function, left atrial size or right ventricular function (Spearmans' correlation coefficients -0.11 [p = 0.31], -0.10 [p = 0.33], and 0.16 [p = 0.14], respectively).

On univariate analysis, there was a significant correlation between RVSP and degree of iron deposition as well as ASCD (p < 0.001, both). The change in FVC% was also significantly associated with RVSP by univariate analysis (p = 0.034), but fibrosis score did not show correlation with RVSP by univariate analysis (p = 0.75) (Table 2).

**Table 2:** Univariate analysis of iron deposition, ASCD, fibrosis score and FVC% with RVSP

Variable	Parameter estimate	P
Iron deposition (n = 114)		< 0.001
Grade 0	Reference	
Grade 1	3.0	
Grade 2	6.6	
Grade 3	18.6	
ASCD (n = 91)		< 0.001
Grade 1 (ASCD < 1.5)	-12.7	
Grade 2 (ASCD ≥ 1.5)	Reference	
Fibrosis score (n = 116)	0.01	0.75
FVC% (n = 113)	-0.2	0.034

Multivariable regression analysis investigating the association of RVSP with iron deposition, ASCD, fibrosis score and FVC% showed that iron deposition maintained the statistically significant association with RVSP (p = 0.02) (Table 2). ASCD did not show a significant association but a trend was observed on this multivariate analysis (p = 0.073) (Table 3). Three initial models were fit with iron deposition, fibrosis score, and the iron deposition by fibrosis score interaction; ASCD, fibrosis, and the ASCD by fibrosis score interaction; FVC%, fibrosis score, and FVC% by fibrosis interaction. There was no interaction between iron deposition and fibrosis score (p = 0.130), nor an interaction between ASCD and fibrosis score (p = 0.22), nor an interaction between FVC% and fibrosis score (p = 0.92). Following this the above multivariable model was fit.

**Table 3:** Multivariate analysis of iron deposition, ASCD, fibrosis and FVC% with RVSP

Variable	Parameter estimate	p
Iron deposition		0.02
Grade 0	Reference	
Grade 1	0.4	
Grade 2	5.0	
Grade 3	14.5	
ASCD		0.073
Grade 1 (ASCD < 1.5)	-6.7	
Grade 2 (ASCD ≥ 1.5)	reference	
Fibrosis	-0.01	0.83
FVC%	-0.12	0.23

## Discussion

PH has been a focus of new investigations due to its potential prognostic and therapeutic implications in IPF patients. Recent studies have demonstrated that severe PH in IPF can occur in the absence of advanced pulmonary dysfunction or hypoxemia [3,15]. As more specific and effective treatment for PH has become available, early diagnosis and treatment of PH could improve the prognosis in the subset of IPF with PH. Right heart catheterization is the gold standard for the diagnosis of PH but is invasive and not widely used in IPF patients. We sought to identify the histologic features that could potentially be the predictors of PH or indicators for further clinical work-up in IPF patients, mainly using the sections of surgical lung biopsies that had been performed for the diagnosis.

It has been thought that PH in IPF develops as a consequence of progressive fibrosis and obliteration of alveoli with vascular changes occurring secondary to the reduction of the pulmonary vascular bed [3,16]. However, this postulation has been questioned as many investigators explored the association of the remodeling of pulmonary vessel in PH associated with IPF. We also have noted that non-fibrotic areas in some lung biopsies of interstitial lung disease (ILD) cases show extraordinary capillary proliferations that were accompanied by clinical PH; they mimicked the changes of pulmonary capillary hemangiomatosis (PCH) with pulmonary veno-occlusive disease (PVOD)-like features in the background (unpublished observations). These cases also had hemosiderin depositions and muscularization of pulmonary arterioles in addition to PCH- or PVOD-like changes, which comprise the features of postcapillary PH. Since the distribution of fibrosis in usual interstitial pneumonia, histopathologic manifestation of IPF, coincides with the distribution of pulmonary veins and venules in the interlobular and interstitial septa of peripheral alveolar acini, these findings could be simply caused by fibrosis. However, one can also postulate that the fibrogenic stimuli involved in IPF might also affect remodeling of postcapillary pulmonary vessels, causing PVOD-like condition.

This led us to hypothesize that these histologic changes may be associated with PH in IPF, possibly independent of lung function or fibrosis. To test this hypothesis, we attempted to measure several histologic parameters in the surgical lung biopsies in the cohort of IPF patients diagnosed over a recent 10-year-period. It was very difficult to quantify these histologic features in an objective, systematic manner. Among these, we found that PCH-like changes by counting the number of endothelial cells on CD34 immunostaining (expressed as ASCD) and hemosiderin deposition assessed by iron stain were most feasible for quantitation in the exploration of this hypothesis. Although smoker's pigments in the alveolar macrophages can mimic hemosiderin, they are typically dustier and distinguishable from refractile, chunky iron pigments.

In our study, iron deposition in IPF was associated with higher RVSP independently of the degree of overall fibrosis or pulmonary function impairment as assessed by fibrosis score on the HRCT scan and FVC on PFT, respectively. Though not reaching the statistical significance, ASCD also showed a trend of association with RVSP on multivariate analysis. FVC% was associated with RVSP on univariate analysis but not on multivariate analysis, however. We analyzed the RVSP data as categorical variable by assigning the value of ≥ 40 mmHg as positive and <40 mmHg as negative for pulmonary hypertension. As was in the analysis using RVSP value as continuous variable, iron score 3 and ASCD score ≥ 1.5 showed the similar findings in both univariate and multivariate analyses (data not shown).

Our study demonstrated HRCT fibrosis score did not show any association with RVSP either on univariate or multivariate analysis, reiterating the conclusion of a previous study; a cross-sectional study of 65 patients with advanced IPF revealed that the extent of pulmonary fibrosis determined on chest HRCT did not predict PH in advanced IPF patients [15]. A recent clinical study also has reported that there is a poor correlation between lung function measures and PH in IPF patients, suggesting that factors other than parenchymal fibrosis may play a role in the pathogenesis of PH [17].

Histopathologic findings of pulmonary vessels in IPF were reported mostly based on vascular abnormalities seen in scarred areas and consist of intimal hyperplasia, fibrosis, and reduplication of the inner elastic lamina in the small muscular pulmonary arteries in fibrotic lobules [18]. However, Colombat et al reported the vascular changes in the architecturally preserved lung tissues as intimal proliferation and fibrosis causing occlusion of venules and small pulmonary veins involving 65% of explant lung specimens from their 26 end-stage IPF cases [16]. Other studies reported increased capillary density and angiogenesis in nonfibrotic lung tissues [19-21]. A greater angiogenic response to IL-8 was detected in UIP lung tissue than in control tissue [22]. A study shows that TGF-beta1-induced and hypoxia-induced VEGF expression by human lung epithelial cells may promote neovascularization, thereby contributing to the repair of injuries to the lung endothelium [23].

The existence of neovascularization in IPF was originally described in 1963 by Turner-Warwick [24]. Heterogeneous vascular remodeling in UIP has been reported by Ebina et al, who also identified increased alveolar septal vascular densities in less fibrotic areas [19]. Several other authors have reported the abnormal vascular remodeling in ILDs although their findings in the literature seemed to be somewhat conflicting [20,25,26]. Chronic inflammation is often associated with fibroproliferation and neovascularization histologically [27]. Inflammation and angiogenesis are also closely related events and temporally coincide [27]. It is possible that neovascularization in ILD is at least partly due to the overlapping properties of many inflammatory, fibrogenic and angiogenic cytokines involved in the pathogenesis of ILDs. A previous study has postulated that an imbalance between the levels of angiogenic and angiostatic chemokines may result in aberrant angiogenesis in both animal models and tissue specimens from IPF patients [28].

A recent clinicopathologic study reported that occlusive venopathy in nonfibrotic lung tissue was observed in 65% of their 26 explanted lungs from end-stage IPF patients [16]. As in our cases, this study also demonstrated that most of these patients had increased alveolar capillary densities with PCH-like changes, hemosiderin depositions and muscularized arterioles [16]. Their statistical analysis showed that the mean pulmonary artery pressure (PAP) correlated with the macroscopic extent of fibrosis, but not with occlusive venopathy [16]. However, this study only included the explanted lungs that would represent the end-stage fibrosis requiring lung transplantation. On the other hand, our cases were mainly composed of surgical biopsies for diagnostic purposes that would include both earlier as well as late stage of IPF cases. This could have been a factor for the differing results between the two studies.

Our retrospective study is mainly limited by the lack of confirmatory right heart catheterization. We used the hemodynamic data obtained by Doppler echocardiography that affords a reasonably good correlation with the right hear catheterization data [29]. One may suspect the iron deposition or increased ASCD in IPF is non-specific, possibly secondary to left heart disease or age-related. In the present study, however; neither echocardial parameters for left heart function nor age of the patients correlated with iron deposition or ASCD, which would argue against such possibilities. We sampled all remaining gross specimens stored in the surgical pathology files for a complete microscopic examination of existing tissues for all surgical biopsies. We also thoroughly examined explanted lungs using multiple representative sections. Some cases still did not have sufficient microscopic fields to assess the changes in nonfibrotic lung tissues, however. We used both FVC% and HRCT fibrosis score to assess the severity of IPF, which should have given a more comprehensive evaluation for the severity than the assessment based on the given biopsy materials as used in a previous study [19].

In summary, our study demonstrated that iron deposition and ASCD, histologic features associated with postcapillary remodeling, are also associated with RVSP in a non-selected cohort of IPF patients. Iron deposition and ASCD were associated or had a trend independent of the severity of disease assessed by FVC% and HRCT. These findings were not related to age or left heart function assessed by echocardiography.

## Conclusion

Our results suggest iron deposition and ASCD as potentially useful morphologic indicators that could lead to the timely diagnosis of PH in IPF patients with appropriate further clinical work-up. This study is limited by the lack of right heart catheterization and further studies would be needed to confirm our finding.

## Abbreviations

PH: pulmonary hypertension; IPF: idiopathic pulmonary fibrosis; UIP: usual interstitial pneumonia; RVSP: right ventricular systolic pressure; BNP: B-type natriuretic peptide; FVC%: percentage of predicted forced vital capacity; HRCT: high resolution computed tomography; ATS/ERS: American Thoracic Society/European Respiratory Society; VVG: Verhoeff-van Gieson; HE: hematoxylin-eosin; ASCD: alveolar septal capillary density; PVOD: pulmonary veno-occlusive disease; PCH: pulmonary capillary hemangiomatosis; ILD: interstitial lung disease

## Competing interests

The authors declare that they have no competing interests.

## Authors' contributions

KHK carried out histopathologic review and drafted the manuscript. FM evaluated the pulmonary function test record in each patient. JHR oversaw the clinical side of IPF diagnosis and evaluated RVSP data in each patient. PWE led the HRCT review for fibrosis score to assign the final score after the review by the two other thoracic radiologists TEH and BJB. PAD carried out the statistical analyses. ESY designed the study, reviewed histopathology and prepared the manuscript. All authors read and approved the manuscript.
